# Hypermobile spectrum disorders symptoms in patients with functional neurological disorders and autism spectrum disorders: A preliminary study

**DOI:** 10.3389/fpsyt.2022.943098

**Published:** 2022-08-24

**Authors:** Veronica Nisticò, Adriano Iacono, Diana Goeta, Roberta Tedesco, Barbara Giordano, Raffaella Faggioli, Alberto Priori, Orsola Gambini, Benedetta Demartini

**Affiliations:** ^1^Dipartimento di Scienze della Salute, Università degli Studi di Milano, Milan, Italy; ^2^“Aldo Ravelli” Research Center for Neurotechnology and Experimental Brain Therapeutics, University of Milan, Milan, Italy; ^3^Dipartimento di Psicologia, Università degli Studi di Milano-Bicocca, Milan, Italy; ^4^Unità di Psichiatria, Presidio San Carlo, Azienda Socio-Sanitaria Territoriale (ASST) Santi Paolo e Carlo, Milan, Italy; ^5^Unità di Psichiatria 52, Presidio San Paolo, Azienda Socio-Sanitaria Territoriale (ASST) Santi Paolo e Carlo, Milan, Italy; ^6^III Clinica Neurologica, Presidio San Paolo, Azienda Socio-Sanitaria Territoriale (ASST) Santi Paolo e Carlo, Milan, Italy

**Keywords:** autism spectrum disorders, functional neurological disorders, conversion disorder, Joint Hypermobility Syndrome, Ehlers-Danlos Syndrome, connective tissue disorder

## Abstract

Autism spectrum disorders (ASDs) and functional neurological disorders (FNDs) share some clinical characteristics such as alexithymia, sensory sensitivity and interoceptive issues. Recent evidence shows that both the disorders present symptoms compatible with a diagnosis of hypermobile Ehlers-Danlos Syndrome and hypermobile spectrum disorders (hEDS/HSD), a heterogeneous group of heritable connective tissue disorders characterized by joint hypermobility, skin hyperextensibility, and tissue fragility. Here we compared the prevalence of hEDS/HSD-related symptoms in a group of patients with FNDs, of people with ASDs without intellectual disabilities, and a non-clinical comparison group (NC). Twenty patients with FNDs, 27 individuals with ASDs without intellectual disabilities and 26 NC were recruited and completed the Self-reported screening questionnaire for the assessment of hEDS/HSD-related symptoms (SQ-CH). We found that 55% of the patients with FNDs, 44.4% of the individuals with ASDs and 30.8% of NC scored above the cut-off at the SQ-CH; SQ-CH scores of both FNDs and ASDs group were significantly higher than the NC group's ones. In conclusion, both ASDs and FNDs individuals present hEDS/HSD-related symptoms in a higher number than the general population. Imputable mechanisms include (i) overwhelming of executive functions with consequent motor competence impairment for ASDs individuals, and (ii) exacerbation of FNDs symptoms by physical injury and chronic pain due to abnormal range of joint mobility. Moreover, we speculated that the amygdala and the anterior cingulate cortex circuitry might be responsible for the imbalances at the proprioceptive, interoceptive, and emotional levels.

## Introduction

Autism spectrum disorders (ASDs) and functional neurological disorders (FNDs) are two relatively common neuropsychiatric conditions, both affecting childhood and adulthood. ASDs refer to a group of neurodevelopmental disorders whose core features concern persistent deficits in social communication and social interaction, and restricted, repetitive patterns of behavior, interests, or activities (1); FNDs consist of symptoms of altered voluntary motor or sensory function that cannot be explained by recognized neurological or medical conditions (1). Despite being apparently separate clinical entities, previous studies showed that ASDs and FNDs share some common clinical and psychopathological features, in terms of alexithymia (difficulties in recognizing one's own emotion at a cognitive level) (2, 3), interoception (the perception of the states and signals coming from within the body) (4–7), and sensory over-responsivity (the excessive or protracted negative response to sensory stimuli), which is known to be a key trait of ASDs (1, 8) and has been recently described also in individuals with a diagnosis of FNDs (9). In a recent paper, we discussed the literature assessing the comorbidity between FNDs and ASDs and showed that the incidence of functional neurological symptoms in a group of adults with ASDs without intellectual disabilities was significantly higher than in a group of healthy neurotypical adults (10). Previous studies also suggest that both the disorders display increased evidence of symptoms compatible with a diagnosis of hypermobile Ehlers-Danlos Syndrome and hypermobile spectrum disorders (hEDS/HSD), (formerly known as Joint Hypermobility Syndrome—JHS) (11–14). The Ehlers–Danlos syndromes (EDS) represent a clinically and genetically heterogeneous group of heritable connective tissue diseases sharing some common features such as joint hypermobility, skin hyperextensibility, and tissue fragility. Since collagen is thoroughly distributed through the body, the manifestations of EDS are multi-systemic and often accompanied by painful sensations. The International EDS consortium now recognizes thirteen subtypes of EDS/HSD (15). Concerning hypermobile EDS (hEDS), despite being the most common EDS subtype, a genetic cause has not been verified yet. Patients with symptomatic joint hypermobility not fulfilling the diagnostic criteria for hEDS are now considered under the umbrella of “hypermobility spectrum disorders” (HSD) (16). Both hEDS and HSD can be associated with functional, extra-musculoskeletal manifestations, such as chronic fatigue, various dysautonomic features, immune system alterations, cognitive disturbances (such as “brain fog”), and psychological distress (17). The case-control study by Bulbena et al. (18) paved the way to several studies investigating the association between EDS and psychiatric disorders. They found that a clinical group composed by hypermobile individuals (i.e., with a positive Beighton Score, a widely used screening test for hEDS) presented a significantly higher rate of panic disorder, agoraphobia, and simple phobia than a group of non-hypermobile individuals (i.e., negative Beighton Score). It is currently known that individuals with hypermobility are up to seven times overrepresented among those with panic or anxiety disorders and exhibited a four times greater probability of manifesting anxiety (19). Moreover, women with hypermobility present higher levels of anxiety than hypermobile men. Other disorders found to be associated with generalized joint hypermobility were depression, schizophrenia, attention deficit hyperactivity disorder, and personality disorders (20).

Aim of this study was to evaluate the prevalence of hEDS/HSD-related symptoms in patients with FNDs, individuals with ASDs without intellectual disabilities, and in a non-clinical comparison group (NC).

## Methods

### Participants

Twenty consecutive patients affected by FNDs and 27 consecutive individuals with ASDs without intellectual disabilities were recruited at the tertiary level neuropsychiatric outpatient clinic of our hospital. Diagnosis of FNDs was made according to DSM-5 diagnostic criteria by a neurologist and a psychiatrist. Diagnosis of ASDs was formulated by a psychiatrist and a psychologist according to DSM-5 criteria (1) and the Module 4 of the Autism Diagnostic Observation Schedule-−2nd version (ADOS-2) (21). The control group was composed by 26 NC individuals, recruited *via* word-of-mouth amongst hospital staff and their acquaintances; their “health state” was assessed through a detailed clinical interview, although they did not undergo any official screening for neuropsychiatric conditions. Exclusion criteria were: (i) age below 18 years; (ii) inability to understand the researcher's instruction or to complete questionnaires because of language difficulties, cognitive disabilities (I.Q. < 70) or dementia; (iii) presence of other severe neurological or medical conditions. The study was approved by the local Ethics Committee. All participants signed an online-written informed consent form.

### Materials

First, demographic and clinical information was obtained by an online questionnaire. Thereafter, each participant completed the Self-reported screening questionnaire for the assessment of Joint Hypermobility Syndrome (SQ-CH) (22), a seven-item instrument including the Hakim and Grahame's five criteria (23) and two additional ones. Each item is presented in a dichotomy format (i.e., participants can only answer “yes” or “no”) and one point is given for each criteria answered affirmatively, hence the Total Score can range from 0 to 7 points. The items consider the entire life of the patient, such as: “As a child you could, or have you ever been able (even now) to place your palms on the ground without bending your knees?”; for this reason, in this study we analyzed possible differences between groups controlling for gender only, and not for age. The SQ-CH has been created as a screening tool to facilitate the HSD diagnosis, which requires a high sensitivity and temporal stability. The SQ-CH is validated only in Spanish: the Italian translation was done by means of a forward and back-translation by an independent translator, blind to the aim of our study. Correlation between the instrument and the widely used Beighton's criteria is high (*r* = 0.9; *p* < 0.001); the cut-off point is established at 3, with a sensitivity of 0.78 and a specificity of 0.24 (22).

### Statistical analysis

Power analysis was conducted with G.Power 3.1.

Statistical analysis was conducted with SPSS 27 (Statistical Package for Social Sciences). Significance level was set at *p* ≤ 0.05, all tests were 2-tailed. First, descriptive statistics were calculated for each group. To assess whether groups were balanced for age and gender, univariate ANOVA and χ^2^ analysis were run, respectively. Second, univariate ANOVA with “Group” and “Gender” as factors and the Total Score of the SQ-CH questionnaire as dependent variable was run; to investigate specific differences between the three groups, Tukey's *post-hoc* analyses were implemented.

## Results

Samples were matched for gender [χ_(2)_ = 5.73; *p* = 0.057] and age [*F*_(2, 70)_ = 2.73; *p* = 0.072]. FND symptoms included: 3 functional weakness; 2 jerks; 1 functional weakness with jerks; 1 functional tremor; 1 functional dystonia; 2 functional gait disorder; 10 Psychogenic non-epileptic seizures (PNES) (Table 1 for further demographic and clinical details about FND group). Psychiatric comorbidity for the ASD group included: 1 Major Depressive Disorder (MDD); 1 MDD and obsessive-compulsive disorder (OCD); 1 MDD and eating disorder; 1 bipolar disorder; 2 anxious-depressive syndrome; 1 Anorexia Nervosa and OCD; 1 dyslexia.

**Table 1:** Demographic and clinical information for patients with FNDs.

**ID**	**Age**	**Sex**	**Diagnosis**	**SQ-CH total score**
FND01	42	F	FM weakness	4
FND02	22	F	PNES	2
FND03	53	F	FMD dystonia	0
FND04	61	F	FMD jerks	4
FND05	60	F	FMD weakness	2
FND06	30	F	PNES	3
FND07	42	F	FMD gait disorder	4
FND08	38	F	PNES	3
FND09	56	M	FMD weakness	3
FND10	38	M	FMD wekness e jerks	2
FND11	28	F	PNES	4
FND12	19	F	PNES	4
FND13	52	F	PNES	4
FND14	39	F	FMD tremor	2
FND15	34	F	PNES	1
FND16	35	F	FMD jerks	2
FND17	61	F	FMD gait disorder	2
FND18	55	F	PNES	2
FND19	55	M	PNES	4
FND20	50	F	PNES	4

*F, Female; FMD, functional movement disorders; FNDs, functional neurological disorders; M, Male; PNES, Psychogenic Non-Epileptic Seizures; SQ-CH, Self-reported screening questionnaire for the assessment of Joint Hypermobility Syndrome*.

**Figure 1:**
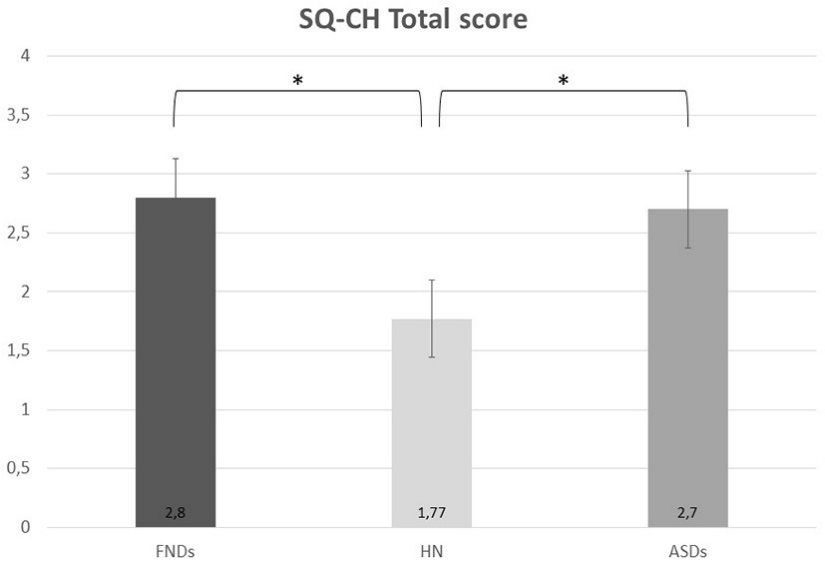
Scores of FNDs, ASDs, and NC groups at the SQ-CH questionnaire. ASDs, autism spectrum disorders; FNDs, functional neurological disorders; NC, non-clinical comparison group. *p < 0.05.

Eleven participants with FNDs, 14 with ASDs, and 8 NC scored above the cut-off for the SQ-CH (Table 2 for further details). At the SQ-CH total score, a significant main effect of group emerged [*F*_(2, 67)_ = 4.03, *p* = 0.022], with both FNDs (*p* = 0.039) and ASDs (*p* = 0.043) patients showing more hEDS/HSD-related symptoms than NC, but no difference between FNDs and ASDs participants (*p* = 0.970; Figure 1). Moreover, a significant main effect of gender emerged [*F*_(1, 67)_ = 5.12, *p* = 0.027], with females (mean score = 2.85, SD = 1.35) scoring significantly higher than males (mean score = 1.63, SD = 1.57) at the JHS questionnaire. No significant interaction effect existed between group and gender [*F*_(2, 67)_ = 1.62; *p* = 0.21].

**Table 2:** Demographic and clinical information for FNDs, ASDs and NC groups.

	**FNDs** **(*N* = 20)**	**ASDs** **(*N* = 27)**	**NC** **(*N* = 26)**
Age, mean (SD)	43.5 (13.06)	40.26 (10.08)	35.96 (10.17)
Gender, M/F	3/17	12/15	12/14
SQ-CH, Y (%)	11 (55%)	14 (51.9%)	8 (30.8%)
SQ-CH, mean (SD)	2.8 (1.2)	2.7 (1.77)	1.77 (1.37)
ADOS-2 Communication, mean (SD)	NA	4 (1.88)	NA
ADOS-2 Reciprocal social interaction, mean (SD)	NA	7.85 (2.7)	NA
ADOS-2 Imagination/creativity, mean (SD)	NA	1.3 (0.67)	NA
ADOS-2 Stereotyped behaviors and restricted interests, mean (SD)	NA	1.44 (1.19)	NA
ADOS-2 Total social communication, mean (SD)	NA	11.85 (4.35)	NA

*ADOS-2, Autism Diagnostic Observation Schedule-−2nd version (Module 4); ASDs, autism spectrum disorders; F, Female; FNDs, functional neurological disorders; NC, non-clinical comparison group; M, Male; N, Numerosity; SD, Standard Deviation; SQ-CH, Self-reported screening questionnaire for the assessment of Joint Hypermobility Syndrome; Y, Yes (no. of participants scoring above the cut-off)*.

## Discussion

The purpose of the study was to assess the presence of hEDS/HSD-related symptoms in a sample of patient with FNDs and a group of individuals diagnosed with ASDs. Results showed that both FNDs and ASDs group scored significantly higher than the NC group at the SQ-CH, hence presenting a higher number of hEDS/HSD-related symptoms; no difference emerged between FNDs and ASDs.

A significant main effect of gender was noted as well, with females scoring significantly higher than males. The latter finding is consistent with the current literature: as previously stated, the incidence of HSD is significantly higher in women than men, although the reason remains poorly understood (16, 17).

With respect to the relationship between ASDs and HSD, Cederlöf et al. (12) performed a population study investigating a possible association between EDS and psychiatric disorders: hypermobile disorders individuals (including EDS) showed a 1.4 increase in relative likelihood for ASDs occurrence; strikingly, EDS alone individuals had a 7-fold higher chance to co-manifest ASDs. Baeza-Velasco et al. (13) found that some features of hEDS/HSD embraced some peculiar traits of the ASD spectrum in terms of social skills, internalizing difficulties, and behaviors. Neurodevelopmental comorbidities frequently co-occur in EDS, especially impaired proprioception (24). Baeza-Velasco et al. (13) claim that, to maintain motor competence despite proprioceptive impairment, executive function may result overwhelmed, leading to some symptoms of ADHD, which is the most common co-occurring psychiatric disorder in ASDs (1). Moreover, pain and dysautonomia, frequently experienced by patients with EDS/HSD, have also been associated with cognitive deficits in attention and concentration. Thus, some characteristics present in EDS/HSD, such as hypermobility, dysautonomia, chronic pain, and proprioceptive impairment, may have consequences in terms of motor, cognitive, and behavioral skills, and may ultimately affect neurodevelopment.

Little is known about the association between FNDs and hEDS/HSD. Kassavetis et al. (11) were amongst the first postulating it, starting from the concept that HSD was linked with psychiatric disorders and other medical conditions whose pathogenesis has not been completely elucidated, including panic disorder, anxiety, irritable bowel syndrome, chronic fatigue syndrome, and fibromyalgia; moreover, they found a 2-fold increase in the incidence of HSD in their cohort of patients with functional movement disorders (FMD) suffering from dystonia. Delgado et al. (14) described the clinical and demographic characteristics of patients with FMD: among other findings, 21% of patients had clinical features suggestive of joint hypermobility, especially those with fixed limb dystonia. It was suggested that aberrant range of joint mobility can lead to physical injury, chronic pain and maladaptive maneuvers, and thus joint hypermobility may be a significant factor in the pathophysiology of fixed dystonia (14, 25). In a recent study by Koreki et al. (26), joint hypermobility was significantly associated with Functional Seizures, also known as psychogenic non-epileptic seizures (PNES), and the association was independent of their anxiety, depression, and other demographic factors such as age, sex, education, and BMI.

Neuroimaging comes into play in ideally explaining these different associations. Neural correlates found to be implicated in HSD are similar to those reported in the above-mentioned psychiatric disturbances, especially in the field of anxiety, alexithymia, and interoception abnormalities: Eccles et al., in a neuroimaging study (27) have found that bilateral amygdala volume was significantly greater in a group of hypermobile patient compared to a group of non-hypermobile individuals; additionally, the same hypermobility group scored higher for interoceptive sensitivity, suggesting a more finely tuned sensory representation of internal bodily signals, and showed a trend toward significantly higher levels of anxiety. These findings suggest amygdala as a likely neural substrate mediating the association between hypermobility, anxiety, and psychosomatic conditions. The hypermobility group showed structural differences within anterior cingulate cortex, a central driver of autonomic arousal and a region implicated in the cognitive control of pain and negative emotions. Mallorquí-Bagué et al. (28) suggest that interoceptive sensitivity mediates the relationship between state anxiety and generalized joint hypermobility. Also, these subjects show increased neural reactivity to sad and angry scenes within brain regions implicated in emotional processing, compared to non-hypermobile subjects, especially in the insular cortex as the common substrate between interoception and emotions. Hence, we might speculate that abnormal signal integration at the level of the circuit involving amygdala, insula, and anterior cingulate cortex might be responsible for the proprioceptive, interoceptive, and emotional imbalances present in the condition, leading to its manifestations. Such speculations, in line with those postulated by Koreki et al. (26), who proposed differences in autonomic control, interoception, and brain structure, associated with joint hypermobility, may predispose patients to Functional Seizures.

Studies on the psychiatric correlates of HSD are in their infancy; this study adds a further piece of evidence to the general framework of the issue, though preliminarily. The major limitations of this study are: the limited sample size; the fact that our control group was not thoroughly screened for neuropsychiatric conditions, but only undergo a detailed clinical interview; the fact that the SQ-CH is not validated in Italian yet; the fact that all data are self-reported and need additional confirmation; further studies should confirm these preliminary data to rule out a chance association. Moreover, future studies with a larger sample size might take into account ASDs comorbidities, to understand whether hEDS/HSD is associated with ASDs subtypes.

## Conclusion

In conclusion, this study provides preliminary evidence that both FNDs and ASDs patients present hEDS/HSD-related symptoms more frequently than the general population; a significant main effect of gender, with women scoring higher than men, has also been noticed. Since the reason underlying the association between hEDS/HSD and psychiatric disorders remains poorly substantiated, future studies are needed to elucidate the potential mechanisms underlying it.

## Data availability statement

The raw data supporting the conclusions of this article will be made available by the authors, without undue reservation.

## Ethics statement

The studies involving human participants were reviewed and approved by ASST Santi Paolo e Carlo. The patients/participants provided their written informed consent to participate in this study.

## Author contributions

VN, AI, DG, RF, and BD contributed to conception and design of the study. VN, AI, RT, and BG collected the data and organized the database. VN and AI performed the statistical analysis. VN, AI, and DG wrote the first draft of the manuscript. RF, AP, OG, and BD revised the manuscript for intellectual content. All authors contributed to manuscript revision, read, and approved the submitted version.

## Funding

This work was partially supported by “Aldo Ravelli” Research Center for Neurotechnology and Experimental Brain Therapeutics, Università degli Studi di Milano, Milano, Italy.

## Conflict of interest

The authors declare that the research was conducted in the absence of any commercial or financial relationships that could be construed as a potential conflict of interest.

## Publisher's note

All claims expressed in this article are solely those of the authors and do not necessarily represent those of their affiliated organizations, or those of the publisher, the editors and the reviewers. Any product that may be evaluated in this article, or claim that may be made by its manufacturer, is not guaranteed or endorsed by the publisher.
